# Physiological characterization of the emergence from diapause: A transcriptomics approach

**DOI:** 10.1038/s41598-018-30873-0

**Published:** 2018-08-22

**Authors:** Vittoria Roncalli, Stephanie A. Sommer, Matthew C. Cieslak, Cheryl Clarke, Russell R. Hopcroft, Petra H. Lenz

**Affiliations:** 1 Pacific Biosciences Research Center, University of Hawai’i at Mānoa, 1993 East-West Rd., Honolulu, HI 96822 USA; 2 Institute of Marine Science, University of Alaska, Fairbanks, 120O’Neill, Fairbanks, AK 99775-7220 USA; 3 Present Address: Department of Genetics, Microbiology and Statistics, Facultat de Biologia, IRBio, Universitat de Barcelona, Av. Diagonal 643, 08028 Barcelona, Spain

## Abstract

Organisms inhabiting high-latitude environments have evolved adaptations, such as diapause to time reproduction and growth to optimize their survival. However, the physiological regulation of the timing of complex life histories is poorly understood, particularly for marine copepods, that diapause at depth. A member of the pelagic community of the sub-Arctic Pacific Ocean, *Neocalanus flemingeri* enters diapause in June. Egg production occurs in winter/spring. In order to characterize the transition from diapause to egg release, females were collected in late September from 400–700 m depth, incubated in the dark at 4–5 °C and sampled for RNASeq at weekly intervals. The diapause phenotype showed down-regulation of protein turnover and up-regulation of stress genes. Activation of the reproductive program was marked by the up-regulation of genes involved in germline development. Thereafter, progress through phases of oocyte development could be linked to changes in gene expression. At 5 weeks, females showed up-regulation of spermatogenesis, indicating that stored sperm had been in a quiescent stage and completed their maturation inside the female. Gene expression profiles provide a framework to stage field-collected females. The 7-week progression from diapause to late oogenesis suggests that females typically spawning in January initiated the reproductive program in November.

## Introduction

Diapause, a developmental program characterized by decreased metabolic activity and increased resistance to cellular stress, constitutes a key survival mechanism for a variety of organisms inhabiting seasonally inimical environments^1,2^. By delaying or arresting development, organisms can regulate the timing of growth, maturation and reproduction to match favorable environmental conditions^1,3,4^. Disruption by environmental change of the conditions that regulate the entrance into and exit from diapause may dramatically impact the continuity in the life cycle of species dependent on it. Advanced warning of population changes is particularly important when the disappearance of a species has cascading effects on other trophic levels^5^. Planktonic copepods of the family Calanidae comprise a group of diapausing crustaceans that might be affected by global warming^6^. These copepods are biomass dominants in high-latitude marine communities, where they are a critical trophic link between primary producers and higher trophic levels^7,8^. A better understanding of their diapause would provide a basis for predicting impacts of environmental change on their annual population cycle.

Calanid copepods support highly productive fisheries in the North Atlantic, Gulf of Alaska and other similar habitats^9–11^. These copepods inhabit environments that are characterized by annual spring phytoplankton blooms, which are typically short in duration (≤1 month) and variable in their timing and amplitude^12,13^. Maturation and reproduction of the copepods is timed such that growth and the accumulation of lipid stores during development occur during the annual peak in primary production. All stages of copepods, including diapausing individuals are a rich and protracted food source for higher trophic levels such as fishes, birds and whales^11^. However, climatic oscillations in combination with global warming are affecting many biotic and abiotic factors in their environment – not only temperature, but also ocean currents, stratification, availability of nutrients and algal bloom dynamics. These may be expected to impact aspects of the biology, physiology and endogenous/exogenous triggers of the diapause program in these copepods^6,10,14,15^. But projecting the effects of abiotic and biotic environmental changes on the life history of a diapausing species requires a solid conceptual framework for understanding of the organism’s ecophysiology and of the mechanisms underlying the developmental program^16,17^. Such a framework is beyond what is currently known for any copepod^10,15^. Thus, the aim of this study was to develop a tool, utilizing the powerful technology of transcriptomics, for elucidating the physiological progression of emergence from diapause to egg release in the copepod *Neocalanus flemingeri*.

No studies on the life cycle of *N*. *flemingeri* have focused on diapause per se, instead what is currently known is based on monthly/bimonthly sampling programs and spring growth experiments^18–20^. *N*. *flemingeri* inhabits the sub-arctic North Pacific and throughout most of its geographic range, it has a single generation per year^18,21^. Mid to late May, the population is found in the upper 100 m and is dominated by the pre-adult copepodite CV stage that are pre-diapause^18,20^. By June, the population disappears from the sub-surface waters, and CV and adult stages accumulate at depth (>300 m). The final molt into the adult stage (CVI) and mating occur at depth, although the specific timing is not well known and may vary between regions^10,18,19,22^. By mid to late summer, the deep population is dominated by adult females, which appear to be in a dormant phase as suggested by the low proportion of ovigerous females between July and September. While the number of ovigerous females starts to increase in October, the highest proportions are observed in December/January. At this time, females show evidence of spawning, and this persists over several months with peak egg production occurring at depth in January^18,19^. Not much is known about the development of the six naupliar stages, however, by March and April early copepodite stages (CI to CIII) become abundant in the upper 100 m and growth is frequently food limited^20^.

Timing is everything: the success of these copepods is dependent on matching the growth phase with the spring phytoplankton bloom. Thus, the production of nauplii occurs during winter/early spring prior to the phytoplankton bloom, while development of the copepodite stages and lipid accumulation occurs during the phytoplankton bloom in April/May. The timing of egg production is in turn dependent on the timing of the reproductive phase. This timing is unknown for *N*. *flemingeri*, since the delay between emergence from diapause and egg release has not been determined. Furthermore, the trigger(s) for emergence remains obscure: the deep environment is highly stable with no fluctuations in temperature or even light that might cue this transition^18,23^. One proposed hypothesis is that diapause termination in calanids is triggered by endogenous changes in either total lipid or lipid composition^24^. However, testing this or alternative hypotheses requires identifying this physiological transition^22^. Here, we characterize the diapause phenotype using gene expression profiling and document the physiological sequence associated with the emergence phase starting with the activation of genes involved in germline development and progressing through different stages of oogenesis and sperm activation.

## Methods

### Collection and transfer to laboratory

*Neocalanus flemingeri* adult females were collected in Prince Williams Sound (station “PWS2”; Latitude 60° 32.1′N; Longitude 147° 48.2′W) during the fall oceanographic cruise of the Seward Line Long-term Observation Program (LTOP) (http://www.sfos.uaf.edu/sewardline/). Samples were collected on September 20^th^, 2015 (7–8 pm) between 700–500 and 500–400 m, using an opening and closing multiple plankton sampler (0.5 m^2^ cross-sectional area; 150 µm mesh nets; Multinet, Hydro-Bios) towed vertically from 700 m depth. Water temperature at depth was 6 °C. Plankton collections were immediately diluted with deep seawater, and stored in the dark at 5 °C prior to sorting. Six healthy *N*. *flemingeri* adult females were sorted then preserved immediately (within 35 minutes of the tow) in RNAlater Stabilization Reagent (QIAGEN)(Wk0). Additional females were live sorted and transferred into carboys and transported in darkness to University of Alaska Fairbanks (UAF) within insulated coolers.

### Experimental design and laboratory incubation

Four days post collection (Sept 24, 2015) the incubation experiment was set up at UAF minimizing exposure to light while completing the sorting and transfer of adult females into 750 mL Falcon tissue-culture flasks. Each tissue culture flask contained four females in 600 ml of seawater that had been collected with Niskin bottles from 600 m depth. The incubation experiment was set up with three replicate tissue-culture flasks per week for a total of ten weeks. The flasks were maintained in an incubator in darkness at 5 °C. Each week, three tissue-culture flasks were randomly chosen and harvested. First, females were assessed for survival and any morphological changes, particularly those associated with gonad maturation in the anterior-dorsal region of the prosome that were visible under a dissecting microscope. We attempted to align our observations with those of the gonad (GS) and oocyte (OS) stages of development previously described for calanoids^25,26^, albeit with imperfect success because our observations were based on live instead of preserved and stained animals. All surviving and healthy females were preserved in RNALater (Ambion) and samples were stored at −80 °C. Flasks were also checked for the presence of eggs and/or nauplii. The first observation of eggs and/or nauplii in the additional Falcon flasks occurred at 7.5 weeks with multiple clutches released between 7.5 and 10 weeks. For the gene expression study, we only included females for the first 7 weeks prior to the beginning of egg release.

### RNA extraction, gene library preparation and RNASeq

Total RNA was extracted from up to 12 individual females from each week using QIAGEN RNeasy Plus Mini Kit (catalog #74134) in combination with a Qiashredder column (catalog #79654) following the instructions of the manufacturer and stored at −80 °C. Total RNA concentration and quality were checked using an Agilent Model 2100 Bioanalyzer (Agilent Technologies, Inc., Santa Clara, CA, USA). For each week, total RNA from six individuals with the highest quality and RNA yields were selected for RNASeq and shipped on dry ice to the University of Georgia Genomics Facility (dna.uga.edu). There, double-stranded cDNA libraries were prepared from total RNA extracted using the Kapa Stranded mRNA Seq kit (KK8420) following manufacturer’s instructions. Briefly, RNA samples were first purified with two oligo-dT selection (polyA enrichment using oligodT beds), and then fragmented and reverse transcribed into double-stranded complementary cDNA. Each sample was tagged with an indexed adapter and paired-end sequenced (PE150 bp) using an Illumina NextSeq 500 instrument using a single High Output Flow Cell. The quality of each RNASeq library (n = 48) was assessed using FASTQC (v1.0.0; Illumina Basespace Labs). For all libraries, FASTX Toolkit (v.2.0.0; Illumina Basespace Labs) was used to: (1) trim the first 9 bp to remove Illumina adapters (TruSeqLT universal primer); (2) remove low quality reads (“Phred” cutoff score ≥30); and (3) set the minimum read length to 50 bp. An average of 5% of low quality reads were removed from each sample, which resulted in 10 to 22 million reads per sample, and an average of 16.8 million reads per sample (Table S1).

### Mapping of short reads and identification of differentially expressed genes (DEGs)

Mapping and statistical analysis was performed using the pipeline described for “Differential expression using a Trinity assembly”^27^. To quantify the expression of each transcript, quality filtered libraries (n = 48) were mapped to an existing *N*. *flemingeri* reference transcriptome (NCBI BioProject PRJNA324453)^28^ using kallisto software (default settings; v.0.43.1)^29^. The reference transcriptome was generated from mRNA sequences from eight adult females representing a range of physiological stages in the transition from dormant to egg-producing females^28^. Briefly, the reference transcriptome generated through the *de novo* assembly of over 400 million reads consisted of 140,841 unique transcripts with an N50 of 1,452 bp.

Differential gene expression analysis was performed using the BioConductor package edgeR^30^ using relative transcript abundances obtained from the mapping step. As implemented by edgeR, prior to statistical testing, RNASeq libraries were normalized using the TMM method (trimmed means of M values), followed by the removal of transcripts with expression levels below 1 count per million (1 cpm) leaving 74,001 transcripts for statistical testing. Differentially expressed genes (DEGs) were identified using the Fisher exact test (as implemented by edgeR) for pair-wise comparisons between individuals from Wk0 and all other weeks (Wk0 vs Wk1, Wk0 vs. Wk2, etc…) with a correction for false discover rate (FDR). Corrected p-values were obtained using the Benjamini-Hochberg method set to p < 0.05. In the results, the magnitude of differential expression is presented as fold change (FC) using the log_2_ ratio of the “experimental” week (Wk1 to Wk7) over Wk0.

### Functional annotation of differentially expressed genes (DEGs)

Functional annotation for DEGs from each comparison (Wk0 vs Wk1, Wk0 vs. Wk2, etc…) were obtained directly from the annotated *N*. *flemingeri* reference transcriptome using SwissProt, Gene ontology (GO) and Kyoto Encyclopedia of Genes and Genomes (KEGG) databases^28^. Briefly, for the annotation the reference transcriptome was searched against SwissProt protein database (*blastx* algorithm) with a maximum E-value of 10^−3^ ^28^. Gene ontology (GO) annotations for biological, molecular processes and cellular component categories as well as KEGG annotations were assigned using UniProt (http://www.uniprot.org/help/uniprotkb) with an E-value of 10^−3^. For the KEGG pathways mapping, genes were searched using KEGG mapper (http://www.genome.jp/kegg/tool/map_pathway1.html). Enrichment analysis was performed for each dataset for up- and down-regulated genes with GO terms against the 59,544 transcripts with assigned GO terms in the *N*. *flemingeri* reference transcriptome^28^. The analysis was implemented using the R package TopGo (v. 2.88.0) with the default algorithm weight01^31^. Fisher exact test and a Benjamini-Hochberg correction with a p-value smaller than 0.05 were used for the statistical significance. It is important to note that in many cases multiple functions (GO terms) are assigned to individual genes.

### Physiological profiling using gene expression

For physiological profiling, gene expression levels were normalized by the number of reads in the sample and transcript length^32^. Relative expression was calculated from the counts generated by kallisto by dividing number of reads per transcript by its length in kilobases and by the number of total mapped reads in millions (RPKM) as implemented by edgeR^30^.

The reproductive signal was analyzed in more detailed given that this process was highly represented among the DEGs between week 0 females and females from all subsequent time points. The *N*. *flemingeri* transcriptome^28^ was searched for all transcripts annotated with GO terms associated with reproduction. Briefly, the AMIGO software GOOSE (December, 2017) was used to search for the “reproduction” GO term (GO:0000003) and its descendants using the LEAD SQL wiki called “find descendants of the node ‘nucleus’ with ‘nucleus’ replaced with ‘reproduction’^33,34^. The list resulted in 9,579 non-redundant genes. Germ cell development (GO:0007281) and oogenesis (GO:0048477) were the two GO terms that were most highly represented among these genes (39 and 31% respectively). These were followed by gonad development (15% of genes, GO:0008406) and eggshell formation (GO: 0030703).

The 9,579 genes involved in reproduction were searched for DEGs, resulting in 1,628 transcripts annotated with one or more “reproductive” GO terms. A heatmap for these DEGs was produced using the *heatmap*.2 function (R software) using the magnitude of the expression difference (log_2_ [fold change]) for each paired comparison between (Wk1 to Wk7) and Wk0. A subset of transcripts from the DEGs identified above was targeted for additional analysis. Based on annotation, selected target genes were identified as being involved in biological/cellular processes that are characteristic for specific processes of the reproductive program. The targeted genes were selected based on studies of the reproduction in model species such as the fruit fly, *Drosophila melanogaster* (http://www.sdbonline.org/sites/fly/aimain/1aahome.htm) and vertebrates^35,36^. For each transcript of interest, average expression in RPKM were obtained for each time point (Wk0 to Wk7) by computing the mean and standard deviation across six biological replicates.

### Expression profile analysis

An independent analysis involved a physiological profiling of each individual female to identify similarity/dissimilarity of relative gene expression among individuals. The aim of this analysis was to establish hierarchical relationships among the individuals based on the similarity of the expression profiles for the genes involved in reproduction independent of time point. Thus, unlike the previous analyses, which focused only on DEGs, the cluster analysis included all the non-redundant genes involved in reproduction (9,579). For each individual, the relative expression (RPKM) was determined for transcripts annotated with GO terms associated with reproduction using kallisto transcript abundance (see above). All females (n = 48) were clustered using the function *hclust* in the base R package, using the average linkage method (UPGMA), and all other default settings^37^.

## Results

### Survival and egg development

**Figure 1:**
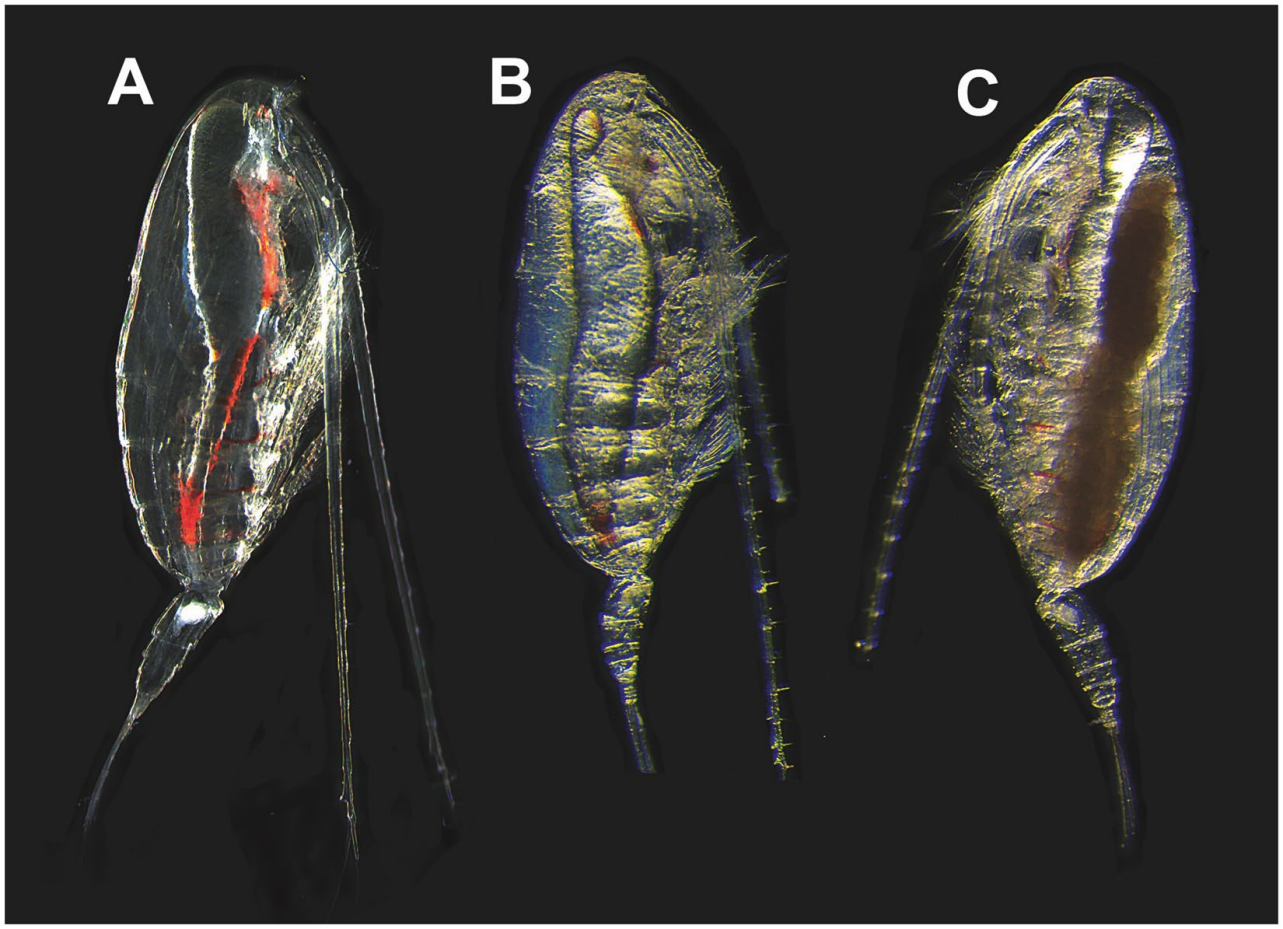
Morphological observations of live *N*. *flemingeri* adult females during experimental incubation. (**A**) *N*. *flemingeri* adult female from Wk1 post collection, showing no evidence of oogenesis but sign of mating with opaque gonopores and seminal receptacles. (**B**) Adult female from Wk4 showing grainy appearance and creamy coloration; evidence of some oocytes in the anterior divernticulum. (**C**) Adult female from Wk7 with oocytes accumulated in lateral oviducts.

Survival was greater than 90% for the first 6 weeks of incubation, and declined to 80% in Wk7. Microscopic observations on live animals, confirmed that females collected from depth (Wk0) were highly transparent with no visible sign of oogenesis. All experimental females showed evidence of mating: opaque gonopores and seminal receptacles (spermatheca) (Fig. 1A). The appearance of the females did not change noticeably during the first two weeks of incubation (Wks 1,2). In Wk3, some females showed the first signs of visible changes including a slight tinting or graininess of the ovary and some differentiation of structure that we equate to gonad maturity stage 1 (GS1)^25^. In Wk4, the ovaries of all females had the grainy appearance indicative of stage GS1. Wk5 and Wk6, females showed more advanced stages of oogenesis and oocytes were more clearly visible, suggesting that females had reached gonad maturity stage 2 (GS2)(Fig. 1B). Furthermore, a few Wk6 females showed signs of oocytes in lateral oviducts that are characteristics of gonad maturity stage 3 (GS3). Oocytes were prominent in the lateral oviducts in Wk7 suggesting that all females had reached gonad maturity stage GS3 or even GS4 (Fig. 1C). Differential gene expression analysis, which extended from Wk0–Wk7, ended just prior to the release of the first clutch of eggs.

### Differential gene expression during emergence from diapause

The number of differentially expressed genes (DEGs) was typically over 4,000 for each paired comparison between Wk0 and all subsequent weeks (Table S2). The first large-scale changes in gene expression were already apparent between Wk0 and Wk1 females. The paired comparison identified 6,254 DEGs (Table S2), which represented 8% of all transcripts expressed at ≥1 count per million (cpm). In general, the number of DEGs increased with length of incubation with the largest number of DEGs occurring between Wk0 and Wk7 females (>10,000 DEGs; Table S2). The number of up- and down-regulated genes among the DEGs was similar (Table S2), as was the magnitude of the differential expression between Wk0 and all other weeks: more than 60% of the DEGs (up- and down-regulated) showed a 2–4 log_2_ fold-change (FC) difference in expression compared with Wk0 individuals (Fig. S1). Approximately 10% of the DEGs were regulated at a 8 log_2_ (FC) or above in the pair-wise comparisons (Fig. S1).

### Functional annotation of DEGs

**Figure 2:**
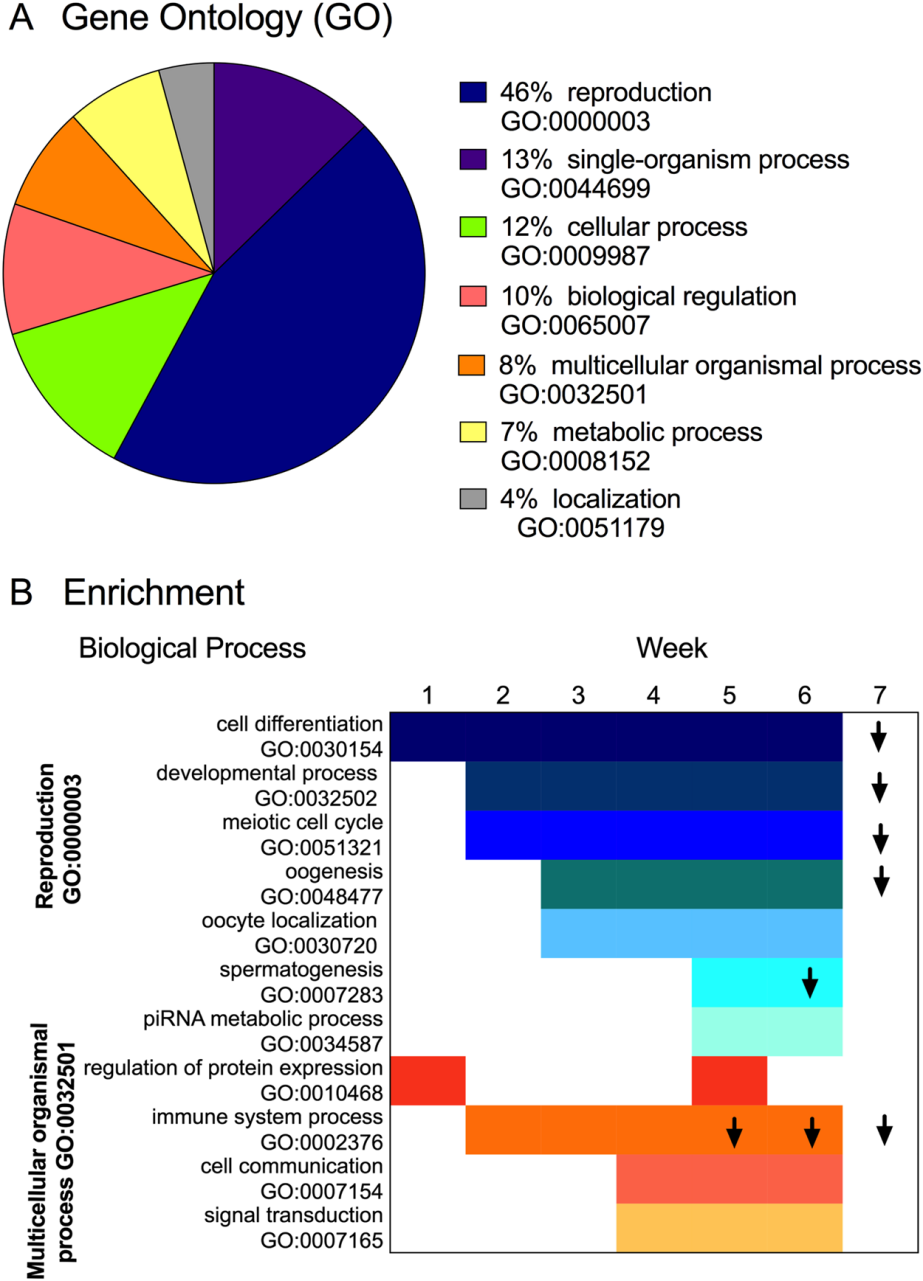
Biological processes represented in the *N*. *flemingeri* gene expression response during the 7-week incubation period following collection of diapausing individuals from depth. (**A**) Pie chart shows proportion of GO terms represented among the DEGs identified for *N*. *flemingeri* adult females in comparisons between Wk0 and Wk1–Wk7 individuals. For the pie chart, DEGs identified in each comparison were obtained independently and annotated. Annotation results for these DEGs were similar across all pair-wise comparisons and were averaged for the pie chart. For each GO term the percentage refers to the ratio between the total number of DEGs annotated within the term and the total number of annotated DEGs obtained for each paired comparison. (**B**) List of enriched GO terms for up-regulated (no arrow) and down-regulated genes (down arrow) in *N*. *flemingeri* adult females for each pair-wise comparison. 11 GO terms were enriched and based on ontology these terms fell within two broad functional GO terms “reproductive process” (blue; GO:0000003) and “multicellular organismal process” (orange; GO:0032501).

Annotation analysis resulted in >80% of the DEGs with a significant hit when blasted against the SwissProt database, with 90% of these with assigned gene Ontology (GO) terms. Within the biological process category (BP), the primary process that distinguished Wk0 females from all others was reproduction (GO:0022414). The percentage of DEGs involved in the “reproductive process” in all pairwise comparisons averaged 40% (range: 37 to 43%). Other biological processes represented among the DEGs included conserved eukaryotic processes such as “single organism process”, “multicellular organismal process”, “biological regulation” and “localization” (Fig. 2A).

More then 70% of the DEGs with gene Ontology (GO) terms retrieved significant KEGG pathway annotations. Most of the DEGs (ca. 75%) were annotated within the metabolism category (M), with nucleotide metabolism, lipid metabolism and energy metabolism (e.g. TCA) being the pathways with the highest number of DEGs (Fig. S2). Fewer than 20% of the DEGs covered transcription and translation pathways within the genetic information processing (GIP) category (Fig. S2). Lastly, only 5% of the DEGs were annotated within the environmental information processing (EIP) category as involved in the membrane transport and signal transduction pathways (Fig. S2). KEGG identified only a small number of DEGs involved in the reproductive process. Searches for KEGG pathways associated with reproduction resulted in the identification of a single pathway “oocyte meiosis” (map0414), which was represented in the reference transcriptome by 14 genes. Target annotation of this pathway (map0414) resulted in the identification of 12 of the 14 expected enzymes (EC #) among the DEGs expressed in Wk1-Wk7 individuals compared with Wk0 (Fig. S3).

Enrichment analysis identified reproduction as the signature process represented among the DEGs in each paired comparison. Out of 11 GO terms that were enriched in the pair-wise comparisons, seven were children GO terms of “reproductive process” (GO:0022414). The remaining four enriched GO terms were children terms of “multicellular organismal process” (GO:002501). Figure 2B, which lists enriched GO terms with their corresponding parent GO terms, shows a temporal progression in the enrichment of the children terms. Within the “reproductive process” group, “cell differentiation” was one of the first processes to be up-regulated, and this GO term was enriched in all pair-wise comparisons between Wk0 and all subsequent weeks. “Developmental process” and “meiotic cell cycle” were among the enriched processes starting in Wk2. However, starting in Wk3, the transcriptomic response became more specific and included GO terms with higher levels of ontology (i.e. more specific biological/cellular function). Thus, in addition to “cell differentiation”, “developmental process” and “meiotic cell cycle”, the GO terms “oogenesis” and “oocyte localization” were identified as enriched among the up-regulated genes. These processes continued to be enriched among the up-regulated genes until Wk6 (Fig. 2B). Starting in Wk7, there was a major shift in the transcriptomic response: GO terms involved in “cell differentiation”, “developmental process”, “meiotic cell cycle” and “oogenesis” were still enriched, however, they were now among the down-regulated genes (Fig. 2B). The timing of this major physiological transition occurred just prior to egg release, which started at 7.5 weeks in this experimental group.

During Wk5, enrichment analysis identified several GO terms involved with male reproduction. The male signals “spermatogenesis” and “piRNA metabolic process” (Piwi-pathway) were enriched among the up-regulated genes (Fig. 2B). A week later (Wk6) “spermatogenesis” had switched its expression and was enriched among the down-regulated genes, while “piRNA metabolic process” was no longer enriched (Fig. 2B).

The “multicellular organismal process” group included four GO terms, which were all associated with the GO term “response to stimulus” at a lower level of ontology. With the exception of Wk1, “immune system process” was enriched in all pair-wise comparisons between Wk0 and Wk2-Wk7. This GO term was initially enriched among the up-regulated genes (Wk4), thereafter it switched and became enriched among the down-regulated genes in the pair-wise comparisons between Wk0 and Wk5, Wk6 and Wk7 individuals (Fig. 2B). “Cell communication” and “signal transduction” both associated with “signaling” process were among the enriched processes starting in Wk3 individuals and continued to be enriched for up-regulated genes until Wk7 (Fig. 2B). An increase in transcriptional activity was identified in Wk1 and Wk5 individuals: the GO term “regulation of protein expression” was enriched among the up-regulated genes during these two weeks.

### Characterization of the diapause phenotype

Compared with females from later weeks, Wk0 individuals had low expression of genes involved in DNA and RNA metabolism (helicase, DNA polymerase, ribosomal protein), protein turnover (serine protease, E3 ubiquitin), mitotic cell cycle (G2 cyclin) and cell differentiation (e.g. innexin 2, neurogenic delta) (Table S3). Expression of many of these genes was significantly higher (~4 log_2_ FC) by Wk1 and remained higher for the rest of the experiment. Moreover, G2 cyclin (5 genes), ribosomal proteins (40S) and serine proteases (3 genes) were among the highest responding genes, with a >10 fold (log_2_ FC) difference in expression between Wk0 and all other individuals.

In addition to the down-regulated genes, the Wk0 diapause phenotype included a number of up-regulated genes. Three transcripts encoding phosphoenolpyruvate carboxykinase (PEPCK) were up-regulated, which is an indication of a higher dependence on anaerobic metabolism. PEPCK relative expression decreased significantly by Wk1 (>3 log_2_ FC) and remained low for the remainder of the experiment (Table S3). We also found significant up-regulation of superoxide dismutase [SOD], glutathione S-transferase Sigma [GST], aldehyde dehydrogenases [ALDH] and cytochrome P450 [CYP450]) in Wk0 females compared with females from Wk2 and later. Relative expression of these genes was high in Wk0 individuals (>30 RPKM), and decreased over time by >2–3 log_2_ (FC) with the lowest expression in Wk7 females (<10 RPKM) (Table S3). Genes encoding heat shock proteins, while well represented in the reference transcriptome (Hsp10, 22, 40, 60, 70, 90 families) did not show significantly higher expression in Wk0 individuals compared with all other weeks.

### Characterization of the reproductive signal

While the enrichment analysis provided an overview of the changes in transcription between Wk0 and subsequent weeks, it provided limited information on the reproductive program between collection and just prior to egg release. In a second analysis, we focused on the specific changes in expression between Wk0 and subsequent time point of all DEGs involved in reproduction.

**Figure 3:**
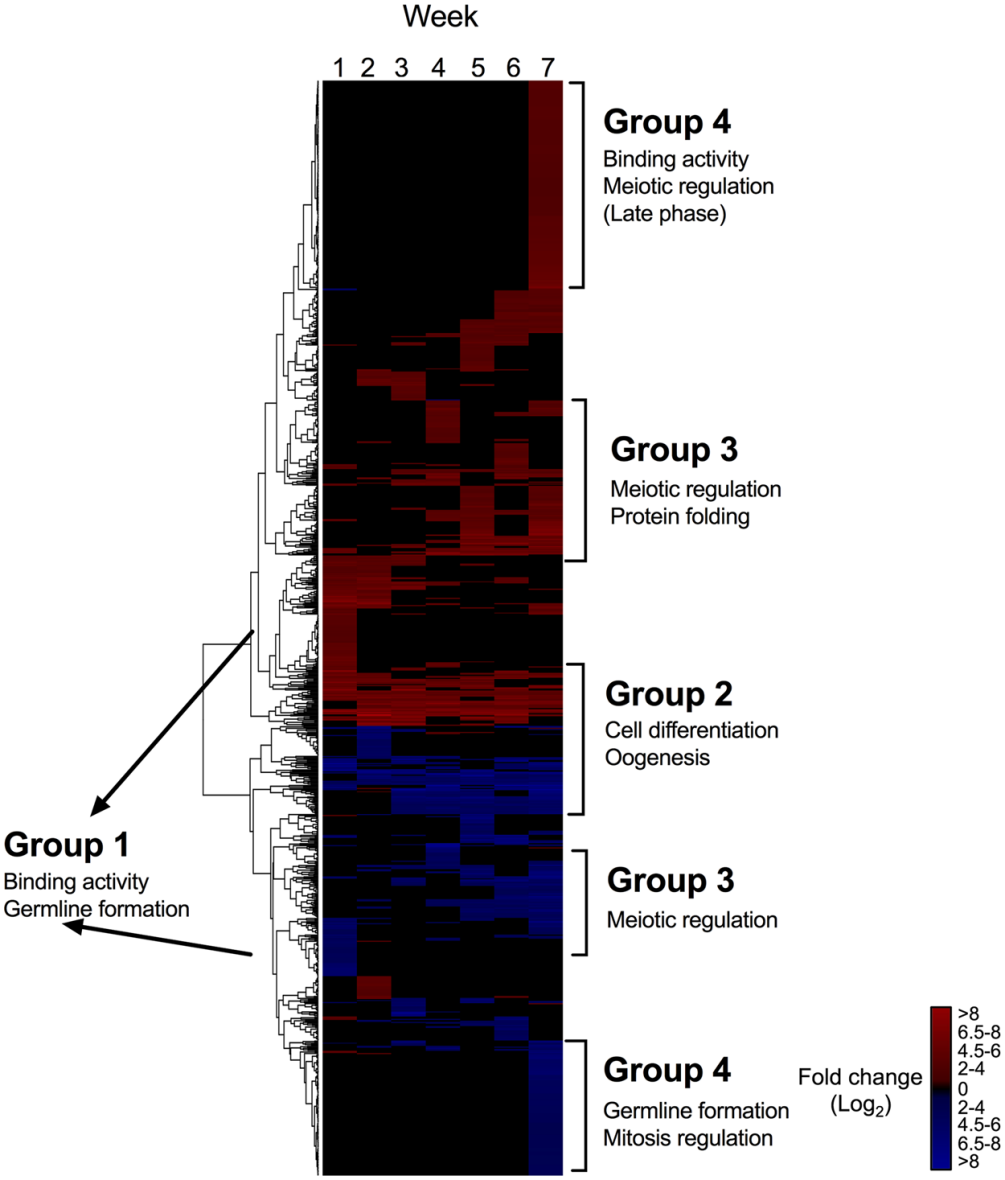
*N*. *flemingeri* reproductive process (GO:0000003). Heat map for DEGs involved in reproductive process (blue color in Fig. 2A) identified in pairwise comparisons between Wk0 females and all other time points (Wk1–Wk7). Color-coding for each gene indicates the magnitude of differential expression (log_2_ FC [Wk_i_/Wk0]). Columns are ordered by time point from Wk1 to Wk7 as indicated by the labels (top). Genes were ordered by similarity of expression pattern as shown by the dendrogram (left).

All pair-wise comparisons searched for genes involved in reproduction yielded 1,628 unique DEGs (Fig. 3). Over 50% of these DEGs were up-regulated in comparison with Wk0 (61%), with the remaining 39% down-regulated (Fig. 3). Based on the heatmap, DEGs could be categorized into four major groups (Fig. 3). Group 1 was characterized by DEGs that were regulated primarily in Wk1 individuals, representing the initial response after collection and indicative of “activation” from dormancy. Among the most highly up-regulated genes (>10 log_2_ FC) were several genes encoding for DNA ligase and G2/mitotic cyclin. Functional annotations represented among this group of DEGs included cell differentiation and more specific processes such as binding activity (ATP- and DNA) and germline development (Fig. 3). Group 2 consisted of genes that were differentially expressed across all weeks and included genes involved in cell differentiation and oogenesis (Fig. 3). Group 3 included a set of mostly up-regulated DEGs that were primarily differentially expressed in the last three weeks (Wk5 to Wk7); and the majority of these (60% of the DEGs) were involved in meiotic regulation and protein folding (Fig. 3). Lastly, Group 4 consisted of DEGs exclusively regulated in Wk7; the up-regulated genes were involved in binding activity (ATP-), meiotic regulation (late phase)(Fig. 3). A set of down-regulated genes were mostly involved in germline development and regulation of mitosis, which is consistent with the identification of these processes as being enriched among the down-regulated genes (see Fig. 2B). Within this group, glutamyl-tRNA transferase and G2/mitotic cyclin were among the highly down-regulated genes (>10 log_2_ FC) in Wk7 females. In addition to these four patterns, a number of genes including some involved in cell cycle and microtubule-organizing center (MTOC) were only highly expressed in females from one or two consecutive weeks. Similarly, genes involved in cell signaling (e.g. kinase) and chromatin rearrangement (e.g. histone) were only up-regulated during the first three weeks.

### Relative expression of genes with specific function in reproduction

An analysis of temporal changes in the expression pattern of target genes with known function during the reproductive program was performed to characterize the developmental progression between Wk0 and Wk7 females. The molecular basis and temporal progression of gene activation during the reproductive program has been characterized in model species including *Drosophila melanogaster* (http://www.sdbonline.org/sites/fly/aimain/1aahome.htm)^35,36,38^. They provided a basis for the selection of genes with known function occurring oogenesis: (1) germline development; (2) previtellogenic development and oocyte differentiation (MTOC); (3) late egg formation and maternal control.

**Figure 4:**
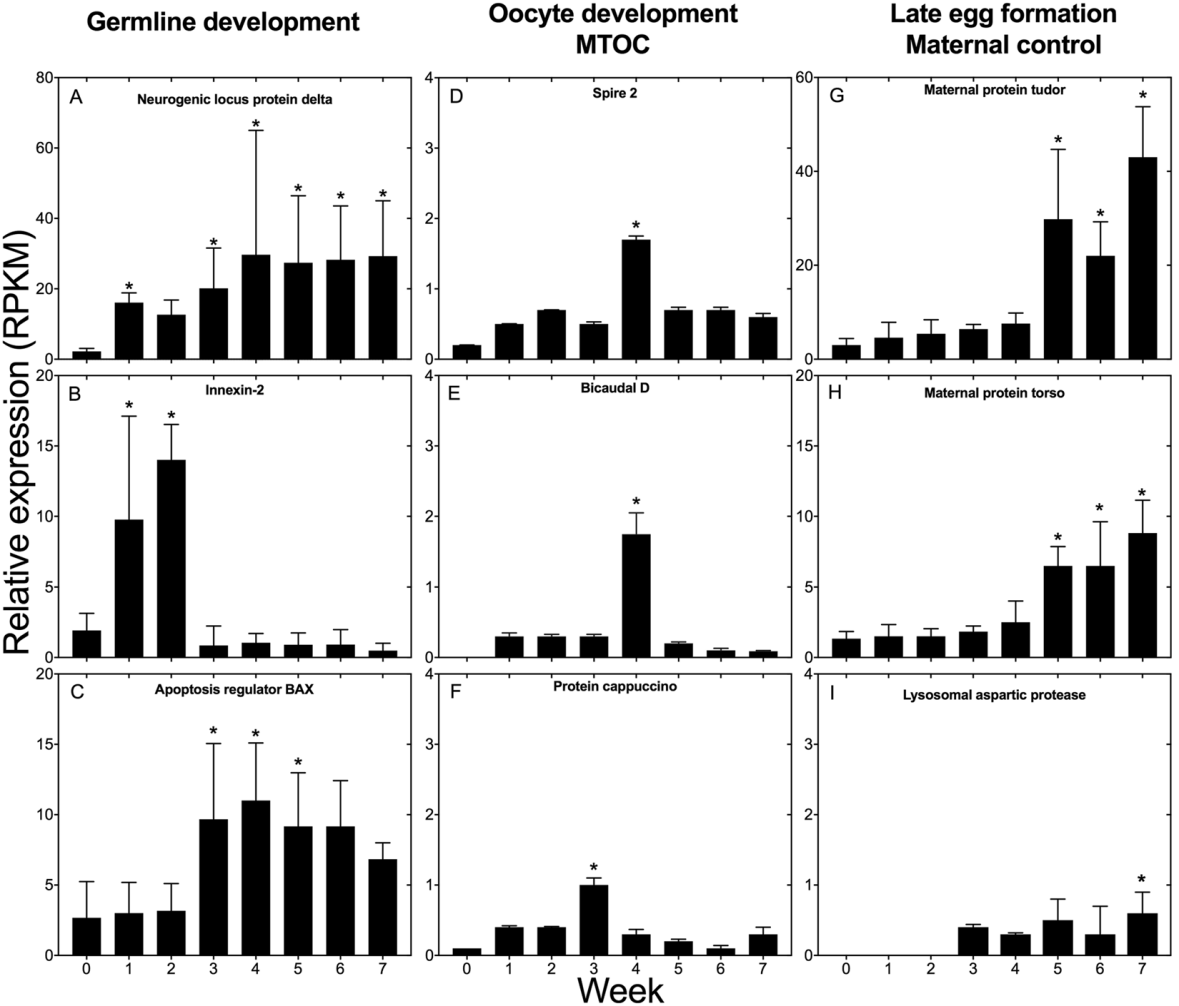
Relative expression of selected *N*. *flemingeri* protein encoding-genes involved in germline development, oocyte differentiation (MTOC), late egg formation and maternal control. Left - Germline development: (**A**) Neurogenic delta, (**B**) Innexin-2, (**C**) Apoptosis regulator BAX. Center - Oocyte differentiation (MTOC): (**C**) Spire, (**D**) Bicaudal, (**E**) Cappuccino. Right - Late egg formation and maternal control: (**G**) Maternal protein tudor, (**H**) Maternal protein torso, (**I**) Lysosomal aspartic protease. For all protein-encoding genes, relative expression is given as RPKM (reads per kilobase per million mapped reads) averaged across six replicate females for each time point (Wk0–Wk7). Error bars are standard deviations of six biological replicates. “*” Indicates differential gene expression between the week sample and Wk0.

The first step during oogenesis involves activation of genes responsible for the development of stem cells into pre-oocytes and oocytes that will develop into mature eggs^38^. We examined the expression of three protein-encoding genes involved in germline development (neurogenic protein delta, innexin 2 and apoptosis regulator BAX) (Fig. 4A–C). Neurogenic locus protein delta showed very low expression in Wk0 females, but by Wk1 expression had increased significantly (≥2 log_2_ FC), and remained up-regulated thereafter until Wk5 (Fig. 4A,B). Relative expression of innexin 2 was also significantly up-regulated by Wk1 (2 log_2_ FC), expression was also high in Wk2, followed by low expression from Wk3 to Wk7 (Fig. 4B). The pattern of expression for the apoptosis regulator protein BAX differed from the two previous genes: the expression was similar in Wk0, Wk1 and Wk2 individuals (Fig. 4C). Significantly higher expression was observed in Wk3 females, and expression remained high through Wk6 (Fig. 4C). The expression pattern of genes encoding neurogenic protein delta and innexin 2 is consistent with the activation of germline development by Wk1, suggesting termination of diapause during the first week following collection.

The next phase of oogenesis is described as previtellogenic development in *D*. *melanogaster*. During this phase oocytes become polarized and a vitelline membrane is secreted and envelops the oocyte (http://www.sdbonline.org/sites/fly/aimain/1aahome.htm)^39^. We examined the expression of three protein-encoding genes that are part of the microtubule-organizing center (MTOC) (spire, bicaudal D, cappuccino). MTOC is responsible of meiotic spindle assembly and involved in oocyte differentiation. Higher expression of genes encoding MTOC proteins is required for the definition of the anterior-posterior axis in the egg, which occurs early in oogenesis after the transition from pre-oocyte to oocyte. The pattern of expression for the three target genes was similar (Fig. 4D–F). Expression of these three genes was very low in Wk0 individuals. There was a consistent, but not significant increase in expression in Wk1 and Wk2, followed by a significant increase during Wk3 for cappuccino, and Wk4 for spire 2 and bicaudal D (Fig. 4D–F). By Wk 4 (cappuccino) and Wk5 relative expression had returned to low (Fig. 4D–F).

The later phases of egg formation are characterized by the increase in the expression of maternal genes expressed upon fertilization. These genes are responsible for polarity of the egg and are typically translated upon fertilization (http://www.sdbonline.org/sites/fly/aimain/1aahome.htm)^35^. Expression was examined for two maternal proteins (tudor and torso) and for the lysosomal aspartic protease, which is involved in the degradation of fat body during vitellogenesis 2 in mosquitoes^40^. Significant up-regulation was found for the two target protein-encoding maternal genes in the later time points (Wk5 to Wk7; Fig. 4G–I). Maternal protein tudor and torso had similar expression patterns with low expression from Wk0 to Wk4 followed by a significant increase during Wk5 (2 log_2_ FC), which persisted through the remaining weeks of the experiments (Fig. 4G,H). As an indicator of the end of vitellogenesis 2, we examined the expression of lysosomal protease; up-regulation of this protein occurrs in *Aedes aegypti* during vitellogenesis 2, coinciding with the decrease of yolk bodies^40^. In *N*. *flemingeri*, the expression of this protein-encoding gene was very low during Wk0 to Wk2, followed by a gradual increase starting in Wk3 that was significant in Wk7 individuals (Fig. 4I).

### Expression of genes associated with male reproduction

**Figure 5:**
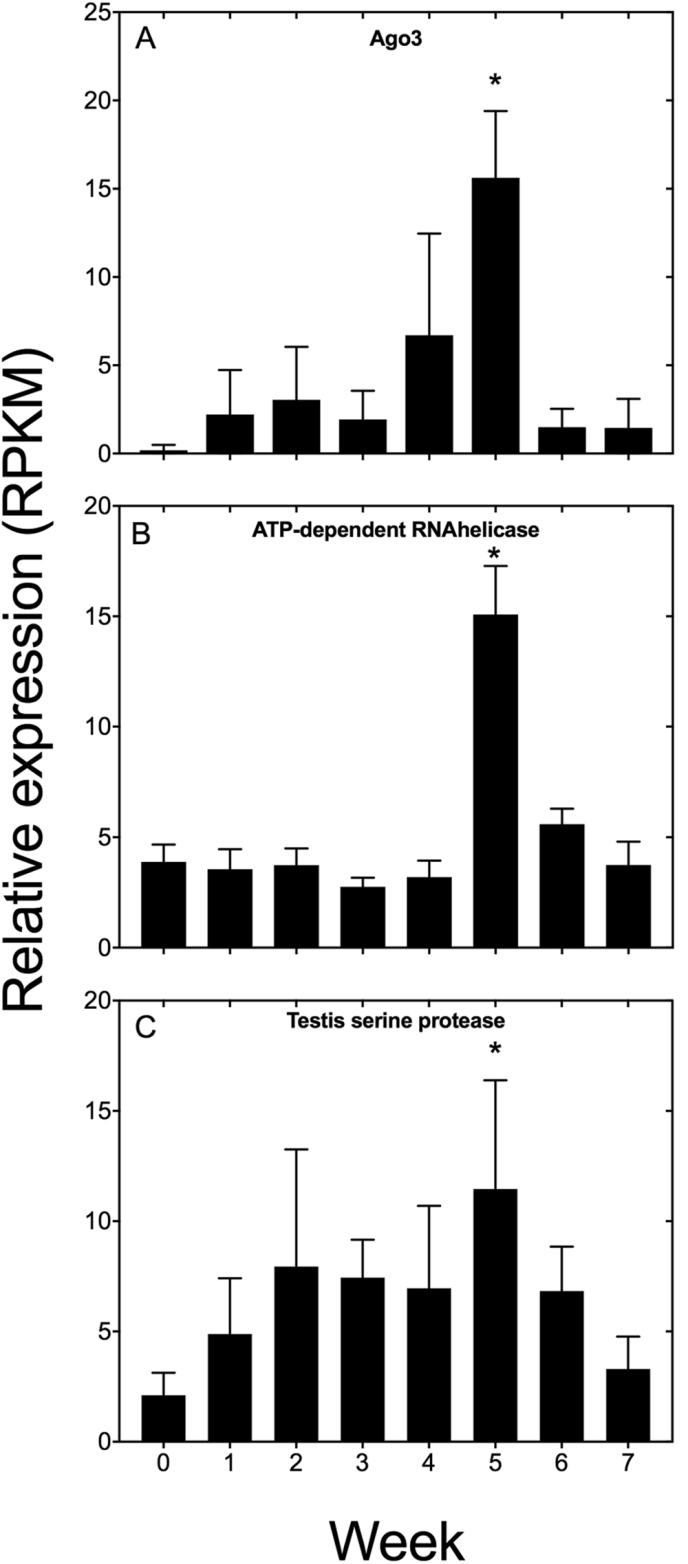
Relative expression of selected *N*. *flemingeri* protein encoding-genes involved in male signal. Relative expression for (**A**) Ago3, (**B**) ATP-dependent RNA helicase and (**C**) Testis serine protease. Relative expression is shown in RPKM as the average expression and standard deviation (error bar) of six adult females for each time point. “*” Indicates differential gene expression between the week sample and Wk0.

A male signal was identified in Wk5 individuals as shown by enrichment among up-regulated genes of the GO terms “spermatogenesis” and “piRNA metabolic process” (See Fig. 2B). More detailed analysis of the specific genes that were differentially expressed showed that several of them are usually considered to be male-specific. In *N*. *flemingeri*, mating occurs before diapause, when males attach spermatophores to the female. Soon after sperm are transferred into the spermatheca where they are stored during the dormant period – insemination and fertilization of the oocytes does not occur until they are released^26,41^. Three protein-encoding genes involved in spermatogenesis were identified among the DEGs in Wk5 (Fig. 5). The first two (Ago3 and ATP-dependent helicase) play key roles in the Piwi pathway, which regulates the repression of transposable elements during spermatogenesis^42^. The third protein-encoding gene, Testis serine protease, is involved in the sperm/egg interaction. Relative expression for Ago3, ATP-dependent RNA helicase and testis serine protease was low for the first weeks but spiked significantly (≥2 log_2_ FC) in Wk5 (Fig. 5), which was followed by a decrease in expression during Wk6 and Wk7 (Fig. 5); for ATP-dependent RNA helicase and testis serine protease there was a 50% decrease between Wk5 and Wk6. A more drastic reduction was observed for Ago3 protein with a 3-fold decrease in expression from Wk5 to the reminder of the experiment.

### Expression profile of reproductive genes

**Figure 6:**
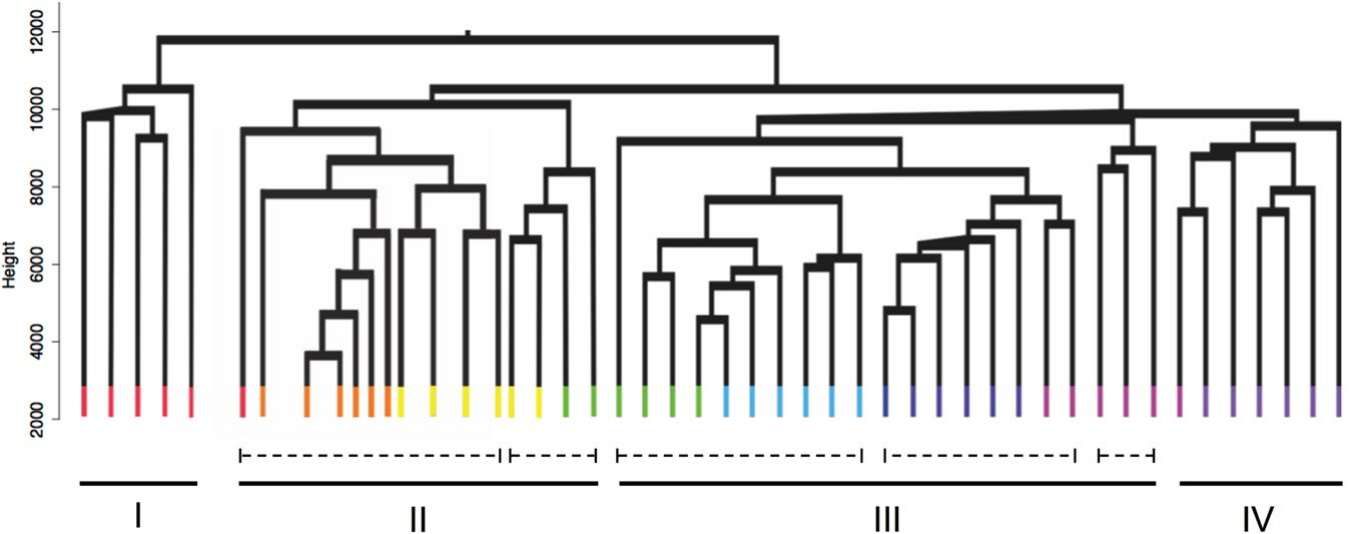
Cluster analysis of *N*. *flemingeri* females during diapause emergence. Dendrogram of all adult females (n = 48) from Wk0 to Wk7 obtained by hierarchical cluster analysis based on 9,579 genes annotated as being involved in reproductive process (GO:0000003). Major clusters (I to IV; solid line) and sub-groups (dashed lines) are shown. Each line represents a single adult female, which is color-coded by time point: Wk0 [red]; Wk1 [orange]; Wk2 [yellow]; Wk3 [green]; Wk4 [light blue]; Wk5 [dark blue]; Wk6 [pink] and Wk7 [dark purple]. Length of the y-axis indicates distance between clusters and individuals.

The relative expression data normalized to RPKM of the annotated reproductive genes were used to generate a physiological profile for each individual female. Females were then clustered by profile similarity. This independent analysis was employed to determine: (1) if females from each time point clustered together; and (2) if clustering could identify developmental phases based on the expression profiles of the reproductive genes. *N*. *flemingeri* adult females (n = 48) segregated into major and minor clusters (Fig. 6). The largest segregation occurred between the first cluster (I) and a group containing three clusters (II–IV). The first cluster included five individuals from Wk0 (red), consistent with the previous analysis that suggests that at collection females were in a different physiological state. The second major cluster (II, 15 individuals) divided in two sub-groups; the first group consisted of 11 individuals with one Wk0 (red), all Wk1 (orange, n = 6) and four Wk2 (yellow, n = 4) females (Fig. 6). The second sub-group in cluster II included the two remaining individuals from Wk2 (yellow, n = 2) and two from Wk3 (green) (Fig. 6). Cluster III had 21 individuals that separated into sub-groups: group 1 included the remaining Wk3 (green, n = 4) and all Wk4 females (light blue, n = 6), group 2 included all Wk5 (dark blue, n = 6) and two Wk6 (pink) individuals, and group 3 included three Wk6 individuals. The 7 individuals in Cluster IV consisted a single female from Wk6 (pink, n = 4) and all from Wk7 (dark purple, n = 6).

The clustering approach indicates that the females were mostly, but not completely synchronous in their physiological progression from Wk0 to Wk7. Furthermore, the clustering suggests three major transitions: the first occurring between Wk0 (red) and Wk1 (orange), the second occurring around Wk3 (green) and the last one between Wk6 (pink) and Wk7 (purple) with the six Wk7 females aligning into a single cluster (IV).

## Discussion

A transcriptomic approach was used to characterize the developmental changes in the transition from diapause (maintenance phase) to egg release in *N*. *flemingeri*. In June, prior to entering diapause, the copepod migrates vertically to 400 m or deeper where it remains for the rest of its life^18^. Molting into the adult and mating occur at depth, which is followed by the disappearance of the males while the females enter a state of arrested/slowed development. Based on morphological observations, egg production normally occurs in January-February^18^. Release of multiple clutches of eggs is followed by the death of the female. The goals of this study were: (1) to compare the copepod diapause phenotype to that described for insects; (2) to link reproductive processes known from *D*. *melanogaster* to stages in copepod oogenesis; and (3) to develop a conceptual framework for mechanistic and ecophysiological studies to investigate timing of life history transitions in copepods with post-embryonic diapause.

Diapause has been predicted for *N*. *flemingeri* based on similarities between its life history and that of a closely related calanid, *Calanus finmarchicus*. In this species the diapause phenotype is characterized by cessation of feeding, antifriction of the digestive tract, low RNA:DNA ratios and a reduction in metabolism and protein turnover^14,43–47^. Upon emergence from diapause, *C*. *finmarchicus* pre-adults undergo a terminal molt, mating occurs at depth and females ascend to the upper 100 m^14,48^. While early oogenesis progresses in the absence of food resources, *C*. *finmarchicus* females lack sufficient lipid reserves to fuel reproduction, and release of the first batch of eggs occurs only after feeding is resumed^49^. The diapause program in *N*. *flemingeri* is more similar to the arctic *Calanus hyperboreus*, which also diapauses in the adult female stage and fuels egg production using stored lipids. However, following egg production in the winter, *C*. *hyperboreus* adult females resume feeding during spring/summer suggesting a multi-year life span with repeated cycles of dormancy^15,49,50^.

*N*. *flemingeri* adult females are non-feeding and reproduction is entirely fueled by stored energy. Upon collection, the females were clear in appearance, and there was no visible evidence of oogenesis, suggesting that the reproductive program was in an arrested state. This was confirmed by low expression of genes involved in reproduction in Wk0 females. In addition, expression profiles of these females showed other similarities with the diapause phenotype described for *D*. *melanogaster* adults^51^. Specifically, Wk0 individuals showed evidence of low metabolism and arrested/slow development as indicated by low expression of genes involved in DNA and RNA metabolism, protein turnover, mitotic cell cycle and cell differentiation, which is also consistent with low RNA:DNA ratios and low protein turnover reported for diapausing *C*. *finmarchicus*. Furthermore, many diapausing insects show a shift to anaerobic pathways as indicated by the up-regulation of phosphoenolpyruvate carboxykinase (PEPCK)^52,53^, which was also observed in *N*. *flemingeri*.

Lengthening of life span and protection from cellular and environmental stress are additional characteristics of the diapause phenotype^54,55^. While there are species-specific differences, genes such as catalase, superoxide dismutase and heat shock proteins are often found to be up-regulated during diapause, presumably to provide protection against oxidative damage and temperature stress. Up-regulation of genes that impart protection from oxidative stress and contribute to homeostasis was observed in *N*. *flemingeri* Wk0 females. However, unlike diapausing insects, which experience wide fluctuations in temperature^1^, heat shock proteins were not up-regulated in Wk0 females. *N*. *flemingeri* spend their diapause phase under constant conditions – the environment in Prince William Sound between 400–700 m is uniformly cold, dark, under high pressure and moderately oxygenated^56^. This expression pattern in *N. flemingeri* suggests that the diapause phenotype includes protection from endogenous cellular stress associated with dormancy.

In our study, the physical manipulation/stress associated with the September collection of *N*. *flemingeri* females from depth effectively terminated diapause (maintenance phase) and initiated the “emergence” program as shown by large differences in gene expression between Wk0 and Wk1 individuals. Clustering analysis segregated Wk1 females from both Wk0 and all other females. Upon activation from the dormant phase, the Wk1 to Wk7 “emergence” time series was dominated by the regulation of genes involved in the reproductive process. The developmental program, which at 5 °C took 7 weeks to complete, was characterized by the sequential up-regulation of genes that control different stages of oogenesis. Egg release in the laboratory-incubated females started in early November, which is two months before the expected peak spawning period in their habitat^18^, leading to the prediction that in nature the termination of diapause and the initiation of the reproductive program for the majority of females occurs in November.

After the first clutch of eggs is released, *N*. *flemingeri* females can produce an additional three to four clutches before being completely spent. However, even before the release of the first batch of eggs, early oogenesis genes (e.g. innexin 2, neurogenic delta, bicaudal, cappuccino) were already significantly down regulated. This down regulation occurred one to two months before the expected release of a female’s last clutch. Thus, once initiated reproduction follows a pre-determined program with early processes associated with oogenesis completed within 6 weeks of the initiation of oogenesis. Thus, once the reproductive program is activated there is little flexibility in the timing of egg release, except through the production of multiple clutches over a 1 to 2 month period.

Morphological, transcriptomic and protein changes during oogenesis have been well characterized in the insect *D*. *melanogaster*^38,51,57^. *D*. *melanogaster* oogenesis consists of fourteen stages that are completed within one week at 25 °C^38^. In copepods, progression through oogenesis consists of five oocyte developmental stages (OS0–OS4), based on changes in oocyte size and location in the female, number and nature of vesicles (yolk, lipid), and appearance of the nucleus^26,58,59^. These histological studies were used in combination with our understanding of reproduction in *D*. *melanogaster* to understand the temporal progression of oogenesis in *N*. *flemingeri* (Table 1).

**Table 1:** Summary of developmental progression during oogenesis in *D*. *melanogaster*, calanid copepods and *N*. *flemingeri*.

Biological process	Genes	Species		
		*D*. *melanogaster*	Calanoid	*N*. *flem*
Budding of the egg chamber from the germarium		Stage 1		Wk0
Germline development Polarization/Terminal follicle cell	Cell-interaction (innexin, nanos, delta)	Stage 2–6	OS0	Wk1
Oocyte polarity-start	Gurken (signaling)	Stage 7	OS1 to early OS2	Wks2–4
Oocyte polarity-end Vitellogeneis 1	MTOC (bicaudal, spire, cappuccino) Epidermal growth factor	Stage 8–10	Late OS2 to OS3	Wks4–6
Vitellogenesis 2 Cytoplasmic dumping	Maternal proteins (tudor, pumilio, torso)	Stage 11	OS3–OS4	Wk7
Development completed Fertilization		Stage 14	OS4	—

Stages of oogenesis (Stage 1–14) in *D*. *melanogaster* and corresponding developmental processes and changes in gene expression are juxtaposed to oocyte development (OS0 to OS4) in calanoid copepods. Proposed correspondence with the developmental progression observed in *N*. *flemingeri* females during emergence from diapause are shown in the last column.

Reference: *D*. *melanogaster*^35,38^, calanoid^18,58,59^.

The first stage (OS0) in the copepod females includes the presence of oogonia and previtellogenic oocytes, which are arrested in the early stages of meiosis I (prophase I)^26^. In *D*. *melanogaster*, the early stages (Stage 2–6) consist of the development of new egg chambers, which are produced from germline and stem cells^51^. These stages are characterized by up-regulation of genes involved in “cell-cell interactions”, which include proteins that regulate signal molecules between germ cells and somatic support cells, and play a role in the regulation of cell adhesion. In *N*. *flemingeri*, up-regulation of genes involved in cell differentiation, germline development (e.g. neurogenic protein delta, innexin 2) and DNA repair suggests that Wk1 females were in the first stage of oogenesis (OS0). Interestingly, this early stage of oogenesis occurs in immature females (stage CV) in other calanid species^60^, which is consistent with the observation that in *C*. *glacialis* females the egg maturation process took only 3 weeks at 0 °C^61^. In contrast, *N*. *flemingeri* appear to delay the reproductive program entering the adult stage at a very early stage of oogenesis, which would explain the long delay between diapause termination and egg release.

In the copepods, the next two stages, OS1 and OS2, are characterized by the progressive movement of oocytes from the ovary to the anterior diverticula and the accumulation of yolk droplets (endogenous source) into the ooplasm (vitellogenesis 1)^26^. In *N*. *flemingeri*, this corresponded to the appearance of light coloration in the anterior diverticula in Wk4 females. In *D*. *melanogaster*, the “mid” stages (stages 7–10) of oogenesis include vitellogenesis 1 and polarization of the oocytes^51^. The overexpression of genes required for defining oocyte polarity such as those associated with the microtubule organizing center (MTOC) occurs during these “mid” stages, which in *N*. *flemingeri* occurred in Wk3 and Wk4 females as indicated by peaks in expression of genes coding for the proteins spire, cappuccino, bicaudal.

The “late” stages of oogenesis (stages 11–13) in *D*. *melanogaster* consist of the transition to and completion of vitellogenesis 2. In the copepods, this occurs during stage OS3, which is characterized by an increase in oocyte size and the accumulation of yolk and lipid vesicles^58,59^. During OS3 oocytes enlarge and yolk and lipid are accumulated in vesicles – the source of yolk and lipids is presumably extra-ovarian^58^. The presence of larger oocytes in the posterior diverticula and oviducts in Wk5-Wk6 females suggests that they were in stage OS3. The change in appearance coincided with the up-regulation of maternal genes (tudor, torso), which is consistent with the late phase of oogenesis in *D*. *melanogaster*^35^. The transition from stage OS3 to OS4 marks the end of vitellogenesis, which occurred between Wk6 and Wk7 as suggested by the clustering pattern of the females. The last stage, which is characterized by a decrease on the fat body lipids, is mediated in mosquitoes by a lysosomal aspartic protease^40^. Up-regulation of a gene encoding for this protease in Wk7 females confirms the completion/near-completion of vitellogenesis and transition to OS4. During the last stage, the nearly mature oocytes complete meiosis I and II as they line up in the oviduct, ready to be inseminated before being released as eggs [reviewed in^26^]. In the current study, the first batches of eggs were observed in the incubation flasks starting at Wk7.5.

**Figure 7:**
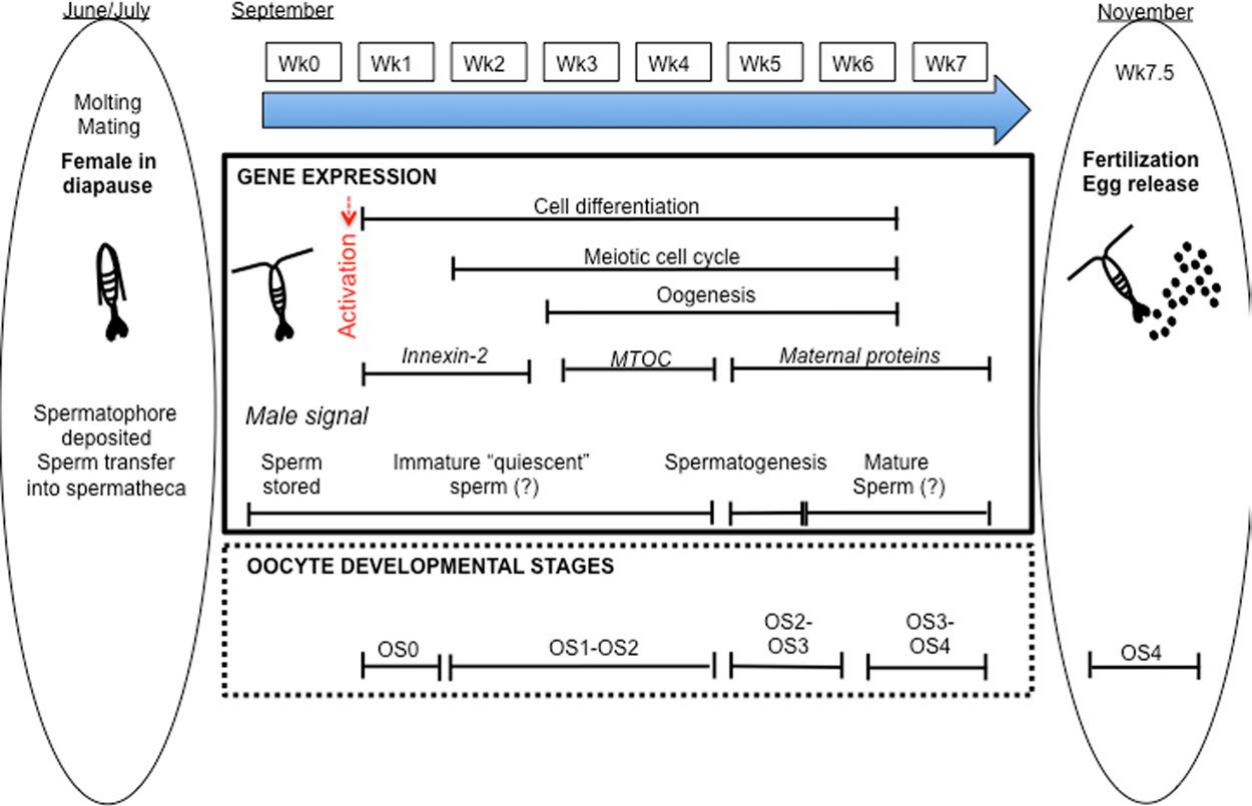
Diagrammatic representation of the proposed developmental progression in *N*. *flemingeri* females from migration to depth (>400 m) to egg release. Central panel with arrow denotes experimental period starting with collection (Wk0, September 20, 2015) and ending in Wk7 (November 5, 2015). Side bars (indicated by ovals) on left (June/July) and right (November) indicate stages prior and after the current experimental period (Wk0–Wk7). Left: late spring/early summer pre-adults CV migrated to 400 m or deeper, molted into into adults, mated, and females entered diapause. Timing based on ecological time series^18^. Prior to diapause, males attach a spermatophore to the female, which is followed by sperm transfer into the spermatheca, and removal of spermatophore^64^. Center: developmental progression between Wk0 and Wk7 showing gene expression (box 1) and stages of oocyte development (box 2). Gene expression (box 1) includes biological processes and target genes (italic), which were enriched at specific time points during the experimental period. Identification of male signal is based on gene expression patterns in Wk5 females. Oocyte development stages are based on macroscopic observation and differential gene expression patterns associated with stage of oogenesis. Nomenclature follows stages based on histological studies of calanoid copepods [reviewed in^26^]. Right: final stages of oocyte development (OS4), which include insemination and egg release. Wk 7.5 corresponds to the presence of the first clutch of egg releases by incubated females in the laboratory.

In contrast to most sperm, which are viable for hours or days, *N*. *flemingeri* females store sperm for months. Based on ecological studies, the males had presumably deposited the spermatophores on the females some time in June-July^18^, while insemination began 4–5 months later in our experiment (early November, Wk7.5). Our collection of *N*. *flemingeri* in September 2015 included extremely few adult males, none of the females had spermatophores attached, and yet females showed evidence of having been mated with (i.e., full spermatheca). While long-term storage of viable sperm (>6 months) by females has been demonstrated for diapausing *Cyclops*^62^ and is likely to occur in *C*. *hyperboreus*^50^, little is known about the cellular mechanisms associated with sperm storage or activation in copepods. The sperm stored in the female spermatheca appear to be in an immature “quiescent” stage (Fig. 7). Enrichment of spermatogenesis and the associated Piwi-pathway in Wk5 individuals suggests sperm activation and final maturation occurred at this time. While the up-regulation of PIWI and Ago3 proteins is involved in sperm maturation in *D*. *melanogaster*, it occurs during the earliest stages of spermatogenesis (prior to meiosis I)^42^. Thus, the role the Piwi-pathway plays in the Wk5 females may be somewhat different and specific to diapausing copepods.

Activation of the sperm appears to be linked to oocyte maturation, since the timing of the spermatogenesis signal (Wk5) coincided with the transition from OS2 to OS3 and the beginning of vitellogenesis 2. Furthermore, the signal was specific to a single week (Wk5) and was followed by significant down-regulation of spermatogenesis in Wk6. In *N*. *flemingeri*, the mature sperm remains viable for another 5 to 8 weeks, since insemination of eggs started about 1.5 weeks later, and continued for another 4–6 weeks, while females released multiple batches of eggs. Interestingly, copepod sperm lack a flagellum and are non-motile^63^, a factor that should lower their energy needs and contribute to their longevity.

Currently, it is not clear whether the up-regulation of male-specific genes originated in the female or in the sperm. If the source was the sperm, then it raises the question of the nature of the cellular machinery required for sperm maturation to occur outside of the male reproductive system. Alternatively, if the female was the source of the up-regulated genes, then it suggests that sperm maturation in *N*. *flemingeri* involves co-ordinated controls from both the male and the female.

## Conclusions

Emergence from diapause was investigated in *N*. *flemingeri* females using global gene expression. Diapausing females were characterized by the down-regulation of genes involved in protein synthesis and protein turnover, while oxidative stress genes were up-regulated. Activation of the reproductive program started approximately 7 weeks prior to egg release. The progression in gene expression provides a framework to classify field-collected females by their stage in the reproductive program. Specifically, gene expression profiling can be used to determine whether females are still in diapause (maintenance phase) or if reproduction has already been activated. Furthermore, high temporal resolution data from field-collected individuals would be instrumental in establishing the degree of developmental synchrony among females and predict the timing of egg release. This type of information is foundational to the goal for identifying environmental and physiological cues involved in the regulation of diapause in this species and other copepods.

## Electronic supplementary material

Supplementary information

Table S3

## Data Availability

The datasets generated and analyzed during the current study are available in the Biological & Chemical Oceanography Data Management Office (BCO-DMO) repository under the Project: Neocalanus Gulf of Alaska [https://www.bco-dmo.org/project/542182]. *N*. *flemingeri* sequence data, as well as the transcriptome Shotgun Assembly (TSA) (GFUD00000000) used as reference, have been submitted to the National Center of Biotechnology Information under the Bioproject PRJNA324453.
