# Supplementary material for: Physiological characterization of the emergence from diapause: A transcriptomics approach
Source: Sci Rep. 2018 Aug 22;8:12577. doi: 10.1038/s41598-018-30873-0 (PMC6105596; doi:10.1038/s41598-018-30873-0)
Supplement: Supplementary file 1 — Supplementary information [file 41598_2018_30873_MOESM1_ESM.pdf]

Physiological characterization of the emergence from diapause: A transcriptomics approach

Vittoria Roncalli[1*], Stephanie A. Sommer[1], Matthew C. Cieslak[1], Cheryl Clarke[2], Russell R. Hopcroft[2] and Petra H. Lenz[1]

[1] Pacific Biosciences Research Center, University of Hawai‘i at Mānoa, 1993 East-West Rd., Honolulu, HI 96822, USA

[2] Institute of Marine Science, University of Alaska, Fairbanks, 120 O'Neill, Fairbanks, AK 99775-7220, USA

Corresponding author: Vittoria Roncalli
Pacific Biosciences Research Center
1993 East-West Rd.
Honolulu, HI 96822
Tel: (808) 956-8003
Fax: (808) 956-6984
E-mail: roncalli@hawaii.edu

*current address: Department of Genetics, Microbiology and Statistics, Facultat de Biologia, IRBio, Universitat de Barcelona, Av. Diagonal 643, 08028, Barcelona, Spain

Table S1. Summary of RNASeq sequencing yields and number of sequences remaining after quality control for *N. flemingeri* adult females. RNASeq was performed on individual females harvested at eight different time points starting at collection from depth (400-700 m; Wk0 to Wk7). RNASeq data were obtained for six females for each time point. For each female, total number of RNASeq reads, number of removed reads for low quality and the number of remaining quality filtered reads are listed. Raw sequences are publicly available through NCBI BioProject PRJNA324453.

SAMPLE	Total # paired ebd reads (F+R)	Quality fitered reads (F+R)	Removed (%)
Collection	17,464,252	16,587,344	5.0
Collection	17,923,464	17,035,224	5.0
Collection	20,210,912	19,244,536	4.8
Collection	19,730,776	18,798,468	4.7
Collection	19,767,462	18,764,386	5.1
Wk 1	16,129,440	15,277,006	5.3
Wk 1	17,325,662	16,420,758	5.2
Wk 1	20,940,104	19,945,588	4.7
Wk 1	19,417,184	18,471,996	4.9
Wk 1	16,761,656	15,823,074	5.6
Wk 2	22,260,920	21,165,876	4.9
Wk 2	19,552,998	18,494,442	5.4
Wk 2	18,234,668	17,299,412	5.1
Wk 2	17,563,826	16,663,660	5.1
Wk 2	17,094,020	16,224,650	5.1
Wk 3	14,269,654	13,584,970	4.8
Wk 3	21,228,176	20,155,530	5.1
Wk 3	16,042,478	15,187,422	5.3
Wk 3	17,479,446	16,605,228	5.0
Wk 3	14,803,794	14,022,320	5.3
Wk 4	23,872,392	*22,508,242*	5.7
Wk 4	18,233,856	17,298,236	5.1
Wk 4	17,885,632	16,940,638	5.3
Wk 4	20,252,666	19,159,522	5.4
Wk 4	23,108,960	21,877,324	5.3
Wk 5	17,320,336	16,312,570	5.8
Wk 5	15,782,800	14,921,072	5.5
Wk 5	22,066,006	20,728,956	6.1
Wk 5	17,057,586	16,203,062	5.0
Wk 5	17,792,476	16,883,718	5.1
Wk 6	20,716,852	19,684,926	5.0
Wk 6	12,810,994	12,213,120	4.7
Wk 6	19,605,330	18,479,234	5.7
Wk 6	23,340,888	22,051,340	5.5
Wk 6	16,099,296	15,103,994	6.2
Wk 7	12,345,606	11,750,070	4.8
Wk 7	12,672,220	12,038,634	5.0
Wk 7	14,805,088	14,037,596	5.2
Wk 7	16,485,430	15,637,432	5.1
Wk 7	11,541,294	*10,993,912*	4.7

Table S2. List of differentially expressed genes (DEGs) identified in pairwise comparisons between Wk0 and all other time points (Wk1 to Wk7) in *N. flemingeri* adult females. The list includes all DEGs (up- and down-regulated) for each paired comparison (Wk0 vs Wk1-Wk7). DEGs were identified using the Fisher exact test ($p < 0.05$) and a multiple comparison correction using Benjamini-Hochberg method (false discovery rate <5%) as implemented by edgeR [30].

**Pairwise comparison**	**DEGs**		
	**Total**	**Up-regulated**	**Down-regulated**
Wk 0 vs Wk 1	6,254	3,885	2,369
Wk 0 vs Wk 2	4,713	3,178	1,535
Wk 0 vs Wk 3	4,537	2,595	1,942
Wk 0 vs Wk 4	6,116	3,424	2,692
Wk 0 vs Wk 5	6,863	3,821	3,042
Wk 0 vs Wk 6	7,031	3,707	3,324

Figure S1. Magnitude of response of differentially expressed genes (DEGs) in *N. flemingeri* adult females. Cumulative distribution of the magnitude of differential expression of up- and down-regulated genes identified for each pair-wise comparison between diapausing females (Wk0) and females harvested at weekly intervals during a 7-week period (Wk1 to Wk7). (**A**) Fold-change ($\log_2$ FC) difference in expression for up-regulated genes. (**B**) Fold-change ($\log_2$ FC) difference in expression for down-regulated genes.

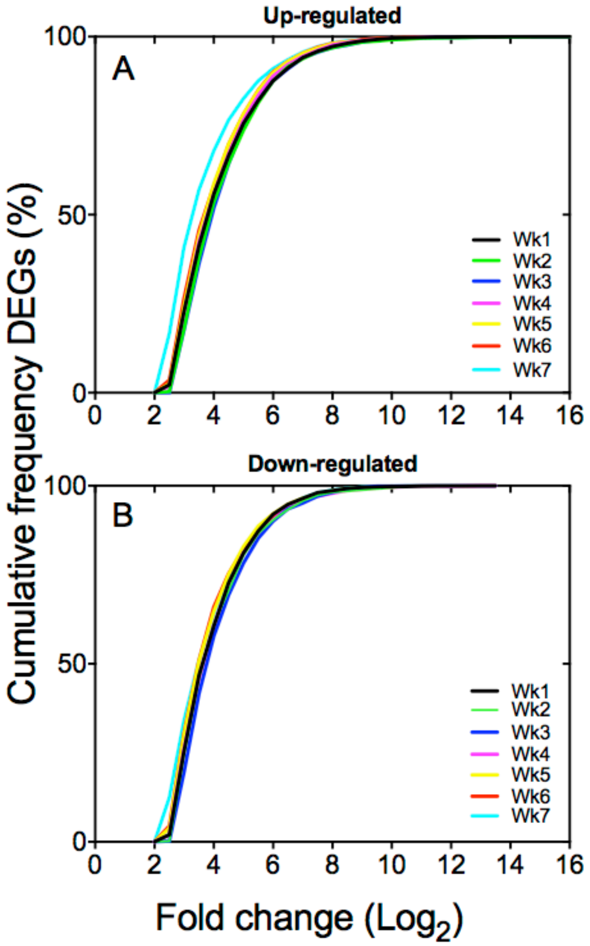

Figure S2. KEGG pathways represented in the differentially expressed genes (DEGs) during the 7-week incubation period following collection of diapausing *N. flemingeri* from depth. Pie chart shows relative proportion of KEGG pathways [31] represented in the DEGs identified in all pair-wise comparisons between Wk0 and Wk1-Wk7 individuals. DEGs identified for each comparison were obtained independently and annotated. Since the KEGG pathways for each comparison were similar, the results shown in the pie chart represent average percentages. KEGG pathway categories: Metabolism (M), Genetic information processing (GIP), Environmental information processing (EIP).

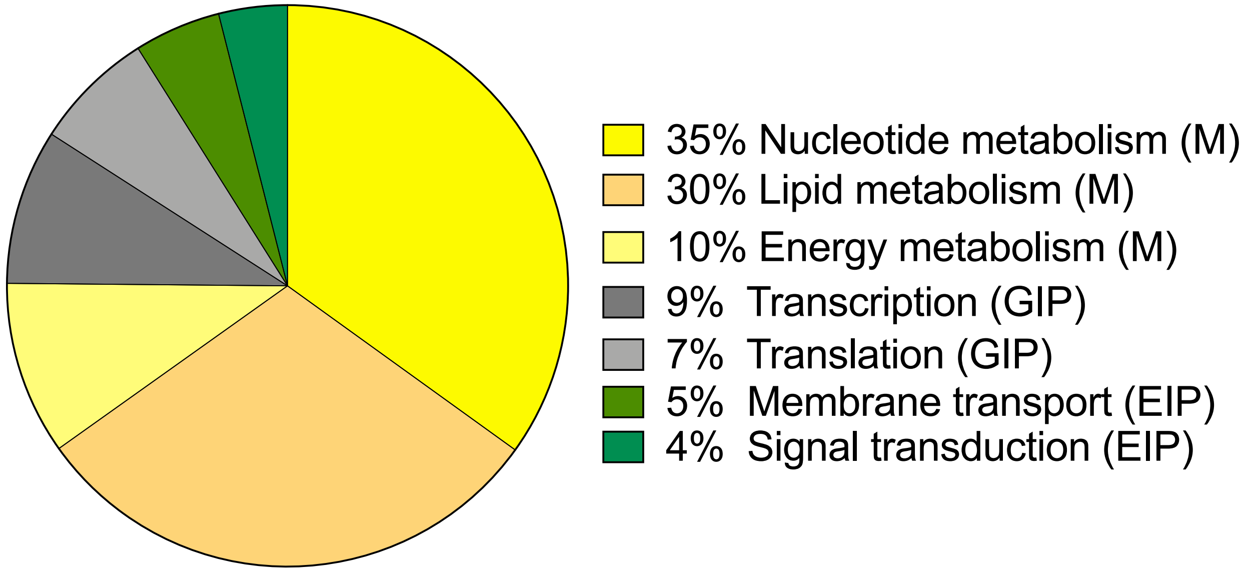

Figure S3. Predicted gene mapping to the oocyte meiosis pathway obtained through KEGG annotation [31]. Oocyte meiosis pathway (map04114) shows the two phases of meiosis (I and II) using *Xenopus* as the model. During meiosis I, homologous chromosomes recombine and then segregate to opposite poles, while the sister chromatids segregate from each other during meiosis II. After the arrest in prophase of meiosis I, resumption of meiosis and progression of the oocyte through two consecutive phases are driven by the maturation-promoting factor (MPF), cyclin B (CycB2/B5) and cell division cycle 2 kinase (Cdc2). Through a negative feedback loop, Cdc2 activates the anaphase-promoting complex (APC), which mediates destruction of CycB2/B5 inducing the oocyte to exit from meiosis I and to progress into interkinesis of meiosis II. To progress into meiosis II, high levels of CycB2/B5 are re-accumulated. APC is inactivated by F-box protein 43 (Emi2) and other components of the CSF (cytostatic factor) such as cyclin E (CycE) and/or high levels of the serine/threonine kinase Mos (MAPK signaling pathway). CSF antagonizes the ubiquitin ligase activity of the APC, preventing the destruction of CycB2/B5 and meiotic exit until fertilization occurs. Target annotation of this pathway for *N. flemineri* identified all the core genes required for meiosis I and II among the DEGs (Wk1-Wk7 individuals compared with Wk0). These genes are highlighted in red. Not surprisingly, considering that the pathway uses *Xenopus* as a model, genes (white boxes) such as the ones regulated by hormones (e.g. insulin like growth factor 1 receptor, progesterone receptor) were absent from the reference transcriptome.

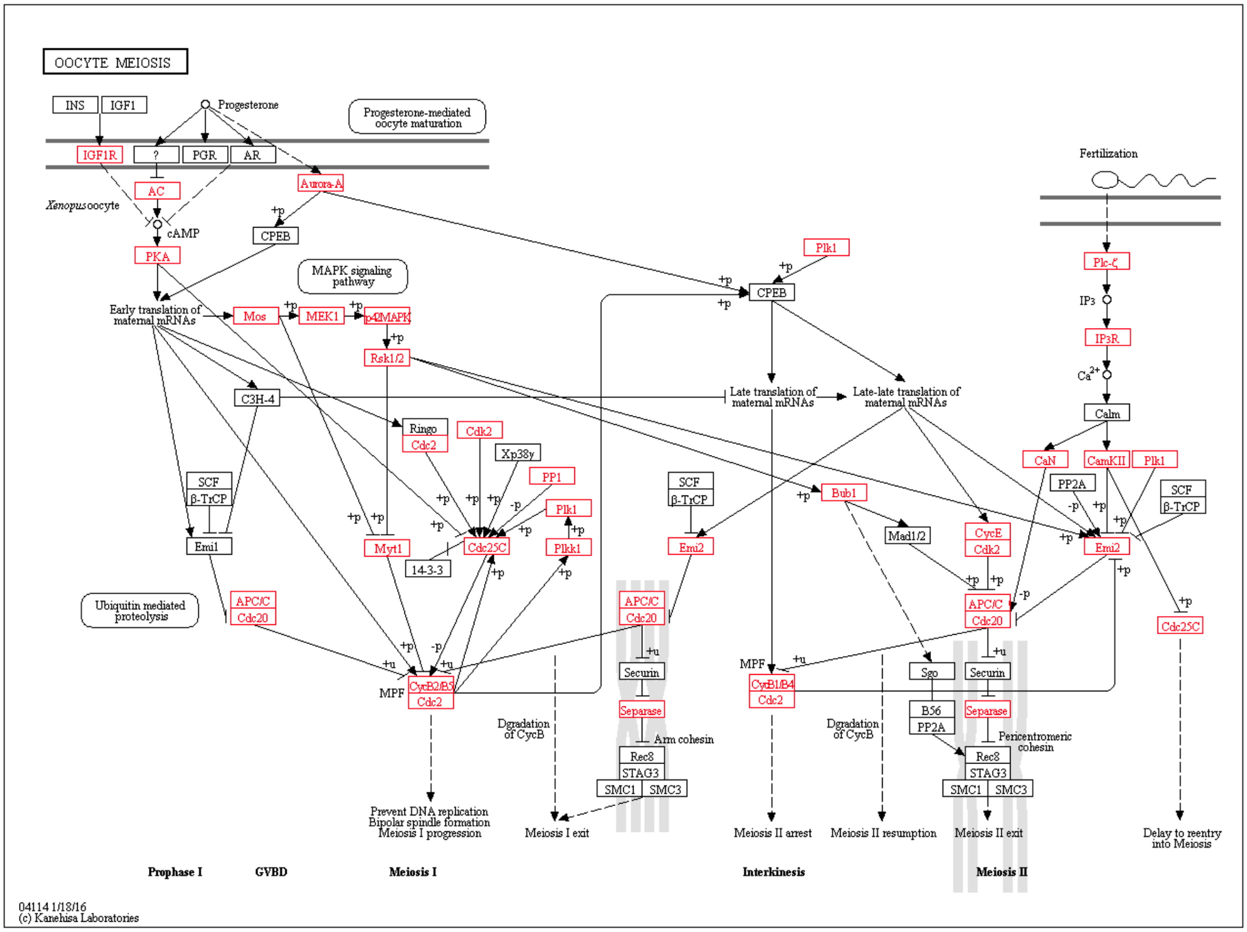
OOCYTE MEIOSIS
INS
IGF1
Progesterone
Progesterone-mediated oocyte maturation
IGF1R
?
PGR
AR
Aurora-A
AC
Xenopus oocyte
cAMP
CPEB
PKA
MAPK signaling pathway
Early translation of maternal mRNAs
Mos
MEK1
p42MAPK
Rsk1/2
C3H-4
Ringo
Cdc2
Cdk2
Xp38γ
PP1
Plk1
SCF
β-TrCP
Emi1
Myt1
Cdc25C
Plkk1
14-3-3
Ubiquitin mediated proteolysis
APC/C
Cdc20
MPF
CycB2/B5
Cdc2
Prevent DNA replication
Bipolar spindle formation
Meiosis I progression
Dgradation of CycB
Meiosis I exit
Securin
Separase
Arm cohesin
Rec8
STAG3
SMC1
SMC3
Plk1
CPEB
Late translation of maternal mRNAs
Late-late translation of maternal mRNAs
SCF
β-TrCP
Emi2
Bub1
Mad1/2
MPF
CycB1/B4
Cdc2
Meiosis II arrest
Dgradation of CycB
Meiosis II resumption
Sgo
B56
PP2A
Securin
Separase
Pericentromeric cohesin
Rec8
STAG3
SMC1
SMC3
Meiosis II exit
Fertilization
Plc-ζ
IP3
IP3R
Ca2+
Calm
CaN
CamKII
Plk1
PP2A
SCF
β-TrCP
CycE
Cdk2
APC/C
Cdc20
Emi2
Cdc25C
Delay to reentry into Meiosis
Prophase I
GVBD
Meiosis I
Interkinesis
Meiosis II
04114 1/18/16
(c) Kanehisa Laboratories
